# Milk Diet

**Published:** 1897-12-11

**Authors:** 


### MILK DIET.

The curative effects of milk diet in many obscure affections are sufficiently well known. In cases of diarrhœa, in cases of ulcer of the stomach, in many cases of innutrition, in some of diabetes, and in plenty of cases of Bright's disease, "milk cures" and "whey cures" have long been used with considerable effect. The milk, however, has mostly been given in such cases for purely empirical reasons. It has been looked upon as a "bland" and soothing diet, notwithstanding the very obvious fact that it often eventuates in the production of hard and massive fæces which appear anything but unirritating. Dr. George Thin is probably right when he suggests that it is not the milk that cures, but the other foods that poison by the abnormal fermentation which they undergo; that it is not the giving of the milk but the withholding of the other food that does the good. In his book on "Psilosis" he has shown how it has happened again and again that patients suffering from that curious disease, who have been doing well on exclusively milk diet for weeks, have experienced a severe relapse so soon as a raw or lightly-boiled egg has been taken, or a cupful of arrowroot has been given; and he infers that in such cases this result is not from any mechanical irritation (which, in fact, could not have been produced by such substances), but has been caused by these substances constituting a pabulum for the development of some form of fermentation which does not occur when milk alone is given. Such an idea derives considerable support from the apparently evil effects which are in some cases seen to follow the use of even a small and apparently insignificant amount of starchy food in cases of rickets. Perhaps the absence of such fermentation in individual cases may also account for the not very uncommon instances in which children have escaped rickets, even though they have been known to partake of starchy food in not inconsiderable quantity, it being not the starch but the fermentation it undergoes in some cases that brews the poison.

The bearing of this point on cases of anæmia and innutrition is of very considerable importance. The well-known views of the late Sir Andrew Clark as to the toxic origin of many cases of anæmia in young women—namely, that they are due to the absorption of some sort of poisonous product resulting from functional derangement of the intestinal canal, and that they should be treated by purgatives—all bear in the same direction, as also does the occurrence of optic neuritis, possibly a mere toxic neuritis, in chlorotic subjects. If all this is granted a good deal follows, and it becomes clear that in the treatment of various conditions of debility and innutrition milk has its true use, not as an extra article of diet, not as a supplement to an already plentiful regimen, but as a means of knocking off the other things. In some of Dr. Thin's cases nutrition began to improve under comparatively small quantities of milk, although they had been steadily failing under a far more "nutritious" diet. This is certainly in accord with experience gained in the treatment of infantile diseases, and the moral is that, if we would gain the full advantage of milk diet in a case of, maybe, obscure disease, the treatment must be milk and nothing else until the experiment has been fairly tried.

